# Effect of fluoride on mechanical properties of NiTi and CuNiTi orthodontic archwires: an in vitro study

**DOI:** 10.1590/2177-6709.26.2.e212020.oar

**Published:** 2021-04-30

**Authors:** Pratap MANE, Chanamallappa R. GANIGER, Renuka PAWAR, Sandesh PHAPHE, Yusuf Ahammed RONAD, Seema VALEKAR, Aditi Aneesh KANITKAR

**Affiliations:** 1 Krishna Institute of Medical Sciences, School of Dental Sciences, Department of Orthodontics and Dentofacial Orthopedics (Malkapur, Karad/MH, India).; 2 Bharati Vidyapeeth Dental College & Hospital, Department of Prosthodontics (Sangli/MH, India).

**Keywords:** NiTi, CuNiTi, Fluoride, Orthodontic wires, Instron machine

## Abstract

**Introduction:**

After debonding, white spot may appear on the area below the bracket, which is the early clinical sign of carious lesion. There is increased caries risk underneath and adjacent to orthodontic bands and brackets, which call for maximum use of caries preventive procedures using various fluoride application methods.

**Objective:**

The aim of the study was to evaluate alterations in the mechanical properties (modulus of elasticity and yield strength) in loading and unloading phases for different orthodontic archwires (nickel-titanium [NiTi] and copper-nickel-titanium [CuNiTi]) when exposed routinely to fluoride prophylactic agents for a predetermined period of time.

**Methods:**

Preformed rectangular NiTi and CuNiTi wires were immersed in fluoride solution and artificial saliva (control) for 90 minutes at 37ºC. After immersion, specimens were tested using a 3-point bend test on a universal testing machine.

**Results:**

There is a significant reduction in the unloading yield strength when the NiTi and CuNiTi wires were exposed to APF gel.

**Conclusion:**

The result suggests that use of topical fluoride agents affect the mechanical properties of the wires, leading to increase in treatment duration. Fluoride prophylactic agents must be used with caution in patients undergoing orthodontic treatment. Injudicious use of these agents may cause corrosive effects on the orthodontic wire surfaces, with alteration in their mechanical properties.

## RESUMO

**Introdução:**

Após a remoção dos braquetes, manchas brancas podem aparecer na área embaixo deles, as quais são o sinal clínico inicial da lesão cariosa. Existe um maior risco de cáries embaixo e ao redor das bandas e braquetes ortodônticos, o que exige a máxima utilização de procedimentos preventivos de cárie, usando diferentes métodos com aplicação de flúor.

**Objetivo:**

O objetivo do presente estudo foi avaliar alterações nas propriedades mecânicas (módulo de elasticidade e resistência ao escoamento), nas fases de carregamento e descarregamento de diferentes fios ortodônticos (níquel-titânio [NiTi] e níquel-titânio com adição de cobre [CuNiTi]), quando expostos rotineiramente a agentes profiláticos fluoretados, utilizados durante um período de tempo predeterminado.

**Métodos:**

Os fios pré-contornados retangulares de NiTi e CuNiTi foram imersos em solução fluoretada e saliva artificial (controle) durante 90 minutos a 37°C. Após a imersão, as amostras foram testadas utilizando-se um teste de flexão em três pontos, em uma máquina universal de testes.

**Resultados:**

Houve uma redução significativa na resistência ao escoamento na fase de descarregamento quando os fios de NiTi e CuNiTi foram expostos ao gel fluoretado.

**Conclusão:**

O resultado sugere que o uso tópico de agentes fluoretados afeta as propriedades mecânicas dos fios, levando a um aumento na duração do tratamento. Os agentes profiláticos fluoretados devem ser utilizados com cautela em pacientes submetidos a tratamento ortodôntico. O uso indiscriminado desses agentes pode causar efeitos corrosivos na superfície dos fios ortodônticos e consequente alteração das suas propriedades mecânicas.

## INTRODUCTION

After debonding, white spot may appear on the area below the bracket, which is the early clinical sign of carious lesion. There is increased caries risk underneath and adjacent to orthodontic bands and brackets, which call for caries preventive procedures using fluoride application methods.

Orthodontic tooth movement results from forces produced by the appliances (wires, brackets, elastics, etc) inserted and activated by the clinician.^1^

NiTi wires are predominantly used in the early stage of orthodontic treatment.

Orthodontists routinely move teeth by attaching brackets to them and activating archwires within the slots of brackets. During space closure, sliding a wire through the slot of a bracket may produce frictional forces, which opposes the treatment plan.

Slot size, surface characteristics of bracket/archwire and the forces used produce friction during orthodontic tooth movement. According to Watanabe et al,^4^ in 2003, the surface roughness of titanium-based archwires increases after exposure to fluoride containing prophylactic agents. Change in the surface characteristics of archwire compromises the sliding of brackets along the archwires.

In 2005, Walker et al^2^ showed that NiTi-based archwires upon exposure to neutral and acidulated fluoride prophylactic agents decreased in mechanical properties. 

Wires containing nickel are used routinely during orthodontic treatment. Oxygen reacts with the surface of all metals to form anoxide surface layer, which protects the metallic surface from corrosion. When friction occurs between the archwires and brackets, the oxide layer dissolves and surface corrosion and pitting take place.

The current study aimed to evaluate changes in the mechanical properties (modulus of elasticity and yield strength), during loading and unloading phases for different orthodontic archwires, when exposed to routinely used fluoride prophylactic agent for a predetermined period of time. Considering the beneficial effects of the prophylactic agents in preventing decalcification of teeth around orthodontic brackets, the objective of this study is to determine whether exposure to these fluoride prophylactic agents causes pitting and corrosion on the surfaces of archwires.

## MATERIAL AND METHODS

The materials used for the study included:

1. NiTi and CuNiTi preformed archwires.
2. Phos-Flur gel and PreviDent 5000
3. Deionized water

The two groups of wires (30 wire specimens) selected were:

» Group I: 0.017 x 0.025-in NiTi archwires (Libral, Okhla Industrial Area, New Delhi, India).

» Group II: 0.017 x 0.025-in CuNiTi archwires (Libral, Okhla Industrial Area, New Delhi, India).

The fluoride agents selected were as follows:

1. Phos-Flur gel (1.1% sodium fluoride acidulated phosphate APF, 0.5% w/v fluoride pH=5.1; Colgate oral pharmaceuticals, New York, USA).
2. PreviDent 5000 (1.1% sodium fluoride neutral agents 0.5% w/v fluoride pH=7; Colgate oral pharmaceuticals, New York, USA).

These fluoride agents were chosen because of their identical methods of application, identical fluoride ion concentrations and differences in pH.

**Figure 1:**
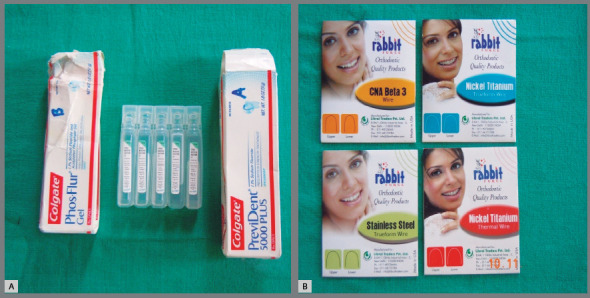
A) Phos-Flur gel, deionized water and PreviDent gel. B) Wires used.

The control solution used was deionized water [dH_2_O].

Each wire specimen was 0.42 X 0.62 X 25mm in dimension, cut from the straight portion of the preformed archwires.

## METHODS

The control group and experimental groups were established.

Control group:

» Ten (n = 10) wire samples consisting of five (n = 5) samples from each wire group, placed in deionized water.

Experimental groups:

» Group I: Ten (n = 10) wire samples consisting of five (n = 5) samples from each wire group, placed in the Phos-Flur gel.
» Group II: Ten (n = 10) wire samples consisting of five (n = 5) samples from each wire group, placed in PreviDent 5000.

Five wires from each group were incubated in 2ml of deionized water at 37°C in an individual plastic 10ml vial or container. This constituted the control sample, with a total of 10 samples. 

Five wires from each group were incubated in 2ml of each fluoride-containing agents in individual plastic containers at 37°C for 90 minutes. These wires constituted the two experimental groups. 

Walker et al,^2^ in 2005, stated that three months of 1 minute daily topical fluoride application or fluoride rinse is equivalent to exposure time of 90 minutes.

Prior to the mechanical testing, the experimental group wires were washed-off with deionized water after removing from the respective solutions and placed in clean and individually coded containers. Wires were randomly selected and were tested using a 3-point bend test on a universal testing machine (model no. 5582J5146, INSTRON; Canton, Mass. USA).

The wires were tested in a chamber with the temperature 37 ± 1°C, which is similar to the oral environment. Each wire was loaded to a deflection of 3.1mm and then unloaded to zero deflection at a cross-head speed of 1mm/min. 

Using the Merlin software program (version 5.43), load in Newtons (N) and deflection in mm were collected every 100 milliseconds for both loading and unloading, for each wire. Using engineering beam theory, Modulus of elasticity (E) and yield strength (YS) in both loading and unloading were calculated using the following formula:

E=L³m/4bd³(GPA)

L = Support span (mm)

b = Width of specimen (mm)

d = Depth of specimen (mm)

m = slope of the straight line position of the loaded or unloaded - deflection curve (N/mm of deflection)

The yield strength (YS) was also calculated as follows:

YS=3PL/2bd²(mPa)

P = Load at the apparent yield point (N)

L = support span (mm)

b = width of specimen (mm)

d = depth of specimen (mm)

ANOVA test was used to do the statistical analysis.

## RESULTS

The distribution of average values for the loading and unloading properties of NiTi wire specimens in deionized water (control) and other experimental fluoride agents is presented in Table 1 and Figure 2A.

**Table 1:** The distribution of average values for the loading and unloading properties of NiTi wire specimens in deionized water (control) and other experimental fluoride agents.

Wire type	Solution	Mean (SD)			
		Loading yield strength (Mpa)	Loading elastic modulus (Gpa)	Unloading yield strength (Mpa)	Unloading elastic modulus (Gpa)
NiTi (n=30)	Deionized water (control) (n=10)	625.5 (38.8)	484.6 (19.7)	334.1 (23.3)	448.6 (2.6)
	Phos-Flur (n=10)	582.7 (61.3)	455.6 (41.4)	258.4 (43.9)	408.2 (45.0)
	PreviDent (n=10)	597.5 (43.2)	454.2 (24.4)	312.8 (33.8)	430.6 (23.2)
	Total (All agents) (n=30)	601.9 (50.4)	464.8 (32.2)	301.8 (46.6)	429.1 (32.8)

Values are presented as mean (SD).

**Figure 2A:**
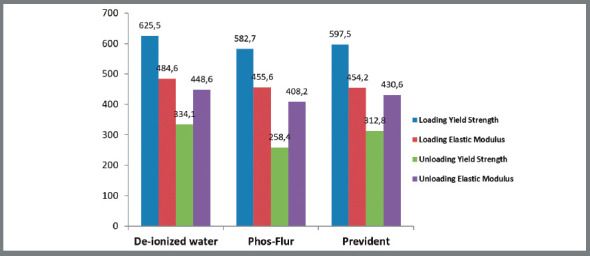
The distribution of average values for the loading and unloading properties of NiTi wire specimens in deionized water ( control ) and other experimental fluoride agents.

Statistical comments:

a) In loading properties, Average yield strength and elastic modulus was not significantly different between various experimental fluoride agents.
b) In unloading properties, Average yield strength and elastic modulus was significantly higher in deionized water, compared to Phos-Flur, but there was no significant difference between deionized water and PreviDent. However, Average yield strength was significantly higher in PreviDent compared to Phos-Flur but the elastic modulus showed no significant difference between Phos-Flur and PreviDent.

The distribution of average values for the loading and unloading properties of CuNiTi wire specimens in deionized water (control) and other experimental fluoride agents is presented in Table 2 and Figure 2B.

**Table 2:** The distribution of average values for the loading and unloading properties of CuNiTi wire specimens in deionized water (control) and other experimental fluoride agents.

Wire type	Solution	Mean (SD)			
		Loading yield strength (Mpa)	Loading elastic modulus (Gpa)	Unloading yield strength (Mpa)	Unloading elastic Modulus (Gpa)
CuNiti (n=30)	Deionized water (control) (n=10)	363.8 (18.1)	369.9 (35.9)	336.9 (7.9)	165.5 (4.7)
	Phos-Flur (n=10)	325.9 (35.4)	325.1 (57.8)	309.5 (35.4)	164.1 (6.9)
	PreviDent (n=10)	357.2 (7.9)	335.8 (24.8)	334.1 (13.6)	165.6 (6.7)
	Total (All agents) (n=30)	348.9 (28.2)	343.6 (44.8)	326.9 (24.9)	165.1 (6.0)

Values are presented as Mean (SD).

**Figure 2B:**
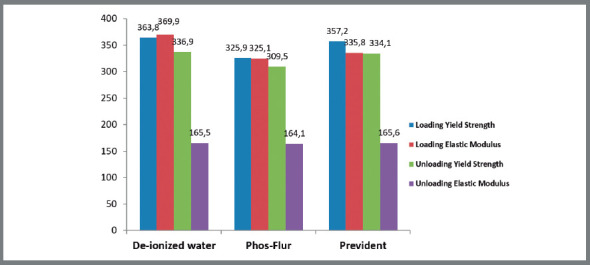
The distribution of average values for the loading and unloading properties of CuNiTi wire specimens in deionized water ( control ) and other experimental fluoride agents.

Statistical comments:

a) In loading properties, Average yield strength was significantly higher in deionized water compared to Phos-Flur, and also it was higher in PreviDent compared to Phos-Flur.
b) Average yield strength was not significantly different between deionized water and PreviDent. The Average elastic modulus was not significantly different between various experimental fluoride agents.
c) In unloading properties, Average yield strength was significantly higher in deionized water compared to Phos-Flur, and it was significantly higher in PreviDent compared to Phos-Flur.
d) The average yield strength was not significantly different between deionized water and PreviDent. The Average elastic modulus was not significantly different between various experimental fluoride agents.

Clinically, corrosion of the CuNiTi wire surfaces increases the friction at the bracket/wire interface, which affects the tooth movement.

## DISCUSSION

In this study, evaluation of two types of wires was done to observe the effect of fluoride agents on unloading mechanical properties (yield strength and modulus of elasticity).

Corrosion of titanium-based archwires^2^ ^-^ ^9^ has been reported due to application of topical fluorides, which suggests that mechanical properties of titanium-based alloys may be altered due to fluoride application, mainly because of hydrogen embrittlement. This further may reduce the clinical efficiency, by increasing the resistance during tooth movement, which can lead to anchorage loss during leveling and aligning, and also during retraction phase. 

According to Toumelin-Chamela et al,^5^ due to hydrogen embrittlement, there is an increase in fracture susceptibility of titanium-based orthodontic wire when kept in fluoride solution. There is a decrease in alloy’s mechanical properties^12^ ^,^ ^13^ due to formation of titanium hydrides, which is reported to form a body centered tetragonal structure^11^.

Sodium fluorides and hydrogen fluoride can cause rapid corrosion^14^. On exposure of titanium-based orthodontic wires to acidulated and neutral topical fluoride agents, hydrofluoric acid (HF) is produced, according to the following equation:

$$H_{3}PO_{4}+\;3\;NAF\to NA_{3}PO_{4}+\;3\;HF$$

The protective oxide layer on the surface is dissolved by hydrofluoric acid causing corrosion and absorption of hydrogen ions from the fluoride solutions, due to high affinity of titanium with hydrogen,^15^ according to the following equation:

$$\begin{array}{l} TI_{2}O_{3}+\;6\;HF\to 2TIF_{3}+\;3H_{2}O\\ \begin{array}{l} \;\\ TIO_{2}+\;4\;HF\to TIF_{4}+\;2H_{2}O\\ TIO_{2}+\;2\;HF\to TIF_{2}+\;H_{2}O \end{array} \end{array}$$

When the fluoride exposure increases, the tensile strength of NiTi alloy decreases to the critical stress level of martensite transformation. Therefore, evaluation of mechanical properties of wire along with corrosion is relevant caused by fluoride agents.

When the NiTi wires were exposed to fluoride for 90 minutes, a decrease in both unloading modulus of elasticity and the unloading yield strength of the wire was found. This is due to the formation of titanium hydride, caused by the hydrogen penetration in the lattice of the NiTi, which alters the lattice’s ability to undergo the unloading phase shift from the martensite form to the austenitic form. In a clinical setting, exposure of NiTi wires to fluoride causes reduction of the tooth movement during unloading stage and also affects the spring-back property of these wires. The effects of fluoride on wire also depends upon the pH and fluoride content of the agent.

When NiTi wires were exposed to APF gel, there was a significant decrease in unloading yield strength, but the unloading modulus of elasticity did not significantly decrease. The mechanical properties are more affected, compared to fluoride agents.

When CuNiTi wires were exposed to APF gel there was a significant decrease in unloading yield strength, compared to control samples.

Due to the presence of copper in CuNiTi wires at the alloy/oxide interface, which avoids the hydrogen penetration, that will reduce the formation of titanium hydride in the lattice structure.

In this study, there was significantly higher yield strength in control PreviDent gel, compared to acidulated Phos-Flur gel; and during the unloading phase, the CuNiTi wires showed significant decrease in mechanical properties. Also, in the present study only the unloading phase was studied, therefore there is a scope for the study of effects of fluoride agents on the mechanical properties.

## CONCLUSION

In the present study, NiTi wires upon exposure to fluoride agents showed significant decrease in mechanical properties. Clinically it is the deactivation forces that cause depletion of mechanical properties of titanium-based archwires. Decrease in the unloading yield strength produces adverse effect, due to the spring-back property of these wires. 

Previous studies have shown that 90 minutes is equivalent to one minute daily application/rinses for a period of approximately three months.^1^ Therefore, in this study the NiTi wires that usually remain in oral environment for more amount of time during leveling and aligning purpose were tested. The fluoride agents that were used are the commonly prescribed oral hygiene agents.

In conclusion, this study suggests that the routinely used fluoride prophylactic agents should be cautiously used in patients during orthodontic treatment because the excess and prolonged use of these agents may cause corrosion of orthodontic wire surfaces along with alteration in mechanical properties, leading to prolonged treatment time.
